# Supplementary material for: Quick sequential organ failure assessment score combined with other sepsis-related risk factors to predict in-hospital mortality: Post-hoc analysis of prospective multicenter study data
Source: PLoS One. 2021 Jul 15;16(7):e0254343. doi: 10.1371/journal.pone.0254343 (PMC8282038; doi:10.1371/journal.pone.0254343)
Supplement: S1 Table — (DOCX) [file pone.0254343.s001.docx]

S1 Table. Comparison of AUCs in each prediction model with multiple imputation

|  | Estimate | 95% CI, lower | 95% CI, upper | p value* |
| --- | --- | --- | --- | --- |
| qSOFA only | 0.641 | 0.596 | 0.686 | Reference |
| qSOFA+Age | 0.668 | 0.623 | 0.713 | 0.026 |
| qSOFA+CCI | 0.655 | 0.611 | 0.699 | 0.060 |
| qSOFA+CFS | 0.681 | 0.638 | 0.725 | 0.005 |
| qSOFA+lactate | 0.706 | 0.660 | 0.753 | <0.001 |
| qSOFA+Age+CCI+CFS+lactate | 0.746 | 0.705 | 0.787 | <0.001 |

*p value: comparison with the AUC of qSOFA-only (model 1)
