# Supplementary material for: Quick sequential organ failure assessment score combined with other sepsis-related risk factors to predict in-hospital mortality: Post-hoc analysis of prospective multicenter study data
Source: PLoS One. 2021 Jul 15;16(7):e0254343. doi: 10.1371/journal.pone.0254343 (PMC8282038; doi:10.1371/journal.pone.0254343)
Supplement: S2 Table — (DOCX) [file pone.0254343.s002.docx]

S2 Table. Comparison of OR and VIF amongst each model

|  | OR | 95% CI, upper | 95% CI, lower | VIF |
| --- | --- | --- | --- | --- |
| **Model 1** |  |  |  |  |
| qSOFA | 2.06 | 1.45 | 2.92 | NA |
| **Model 2** |  |  |  |  |
| qSOFA | 1.91 | 1.34 | 2.72 | 1.01 |
| Age | 1.03 | 1.01 | 1.04 | 1.01 |
| **Model 3** |  |  |  |  |
| qSOFA | 1.69 | 1.17 | 2.44 | 1.06 |
| CFS | 1.22 | 1.11 | 1.34 | 1.06 |
| **Model 4** |  |  |  |  |
| qSOFA | 2.07 | 1.46 | 2.95 | 1.00 |
| CCI | 1.05 | 0.99 | 1.12 | 1.00 |
| **Model 5** |  |  |  |  |
| qSOFA | 1.43 | 0.97 | 2.09 | 1.10 |
| Lactate | 1.19 | 1.13 | 1.26 | 1.10 |
| **Model 6** |  |  |  |  |
| qSOFA | 1.09 | 0.73 | 1.64 | 1.17 |
| Age | 1.03 | 1.01 | 1.04 | 1.14 |
| CFS | 1.20 | 1.08 | 1.33 | 1.19 |
| CCI | 1.03 | 0.96 | 1.1 | 1.04 |
| Lactate | 1.23 | 1.16 | 1.3 | 1.16 |

The square root of the variance inflation factor indicates how much larger the standard error increases compared to if that variable had 0 correlation to other predictor variables in the model.

Abbreviation: CCI, charlson comorbidity index; CFS, clinical frailty scale; CI, confidence interval; OR, odds ratio; qSOFA; quick sepsis-related organ failure assessment; VIF, variance inflation factor
