# Supplementary material for: Quick sequential organ failure assessment score combined with other sepsis-related risk factors to predict in-hospital mortality: Post-hoc analysis of prospective multicenter study data
Source: PLoS One. 2021 Jul 15;16(7):e0254343. doi: 10.1371/journal.pone.0254343 (PMC8282038; doi:10.1371/journal.pone.0254343)
Supplement: S3 Table — (DOCX) [file pone.0254343.s003.docx]

S3 Table. Subgroup analysis based on the microbiological evidence of the infection

**Confirmed infection (n=866)**

|  | **estimate** | **95% CI, lower** | **95% CI, upper** | **p-value** | **threshold** | **sensitivity** | **specificity** | **ppv** | **npv** |
| --- | --- | --- | --- | --- | --- | --- | --- | --- | --- |
| **qSOFA only** | 0.632 | 0.583 | 0.680 | 1.000 | 0.165 | 0.571 | 0.617 | 0.223 | 0.882 |
| **qSOFA+Age** | 0.653 | 0.603 | 0.703 | 0.096 | 0.164 | 0.614 | 0.609 | 0.232 | 0.891 |
| **qSOFA+CCI** | 0.645 | 0.597 | 0.693 | 0.106 | 0.149 | 0.636 | 0.588 | 0.229 | 0.893 |
| **qSOFA+CFS** | 0.669 | 0.621 | 0.717 | 0.018 | 0.167 | 0.607 | 0.653 | 0.252 | 0.896 |
| **qSOFA+lactate** | 0.702 | 0.653 | 0.751 | 0.000 | 0.160 | 0.600 | 0.745 | 0.312 | 0.906 |
| **qSOFA+Age+CCI+CFS+lactate** | 0.738 | 0.693 | 0.782 | 0.000 | 0.154 | 0.679 | 0.669 | 0.284 | 0.915 |

**Non-infection (n=80)**

|  | **estimate** | **95% CI, lower** | **95% CI, upper** | **p-value** | **threshold** | **sensitivity** | **specificity** | **ppv** | **npv** |
| --- | --- | --- | --- | --- | --- | --- | --- | --- | --- |
| **qSOFA only** | 0.496 | 0.330 | 0.661 | 1.000 | 0.125 | 0.800 | 0.257 | 0.133 | 0.900 |
| **qSOFA+Age** | 0.591 | 0.406 | 0.776 | 0.455 | 0.138 | 0.700 | 0.529 | 0.175 | 0.925 |
| **qSOFA+CCI** | 0.541 | 0.344 | 0.738 | 0.639 | 0.125 | 0.500 | 0.614 | 0.156 | 0.896 |
| **qSOFA+CFS** | 0.593 | 0.409 | 0.777 | 0.411 | 0.120 | 0.700 | 0.514 | 0.171 | 0.923 |
| **qSOFA+lactate** | 0.522 | 0.291 | 0.753 | 0.848 | 0.117 | 0.500 | 0.614 | 0.156 | 0.896 |
| **qSOFA+Age+CCI+CFS+lactate** | 0.617 | 0.421 | 0.813 | 0.344 | 0.142 | 0.500 | 0.714 | 0.200 | 0.909 |

Abbreviation: CCI, charlson comorbidity index; CFS, clinical frailty scale; CI, confidence interval; qSOFA; quick sepsis-related organ failure assessment
