# Supplementary material for: Quick sequential organ failure assessment score combined with other sepsis-related risk factors to predict in-hospital mortality: Post-hoc analysis of prospective multicenter study data
Source: PLoS One. 2021 Jul 15;16(7):e0254343. doi: 10.1371/journal.pone.0254343 (PMC8282038; doi:10.1371/journal.pone.0254343)
Supplement: Attachment — Submitted filename: ResponsetoReviewers.docx [file pone.0254343.s004.docx]

**May 20, 2021**

**Dr Juan Carlos Lopez-Delgado, MD, PhD
*PLOS ONE***

**Dear Editor:**

Thank you for inviting us to submit a revised draft of our manuscript to the *PLOS ONE*. We appreciate the time and effort you and each of the reviewers have dedicated to providing insightful feedback on ways to strengthen our paper.

Below, we have addressed the questions and comments delivered in your previous letter. We look forward to your editorial decision.

Sincerely,

Atsushi Shiraishi

Emergency and Trauma Center, Kameda Medical Center,

929 Kamogawa, Chiba, 296-8602, Japan

Email: [siris.accm@tmd.ac.jp](mailto:rueno-tky@umin.ac.jp)

Tel: (+81) 4-7092-2211

**Journal Requirements:**

**1. Please ensure that your manuscript meets PLOS ONE's style requirements, including those for file naming.**

[Response]

We have edited the file names as per the journal’s guidelines.

**2. In the ethics statement in the manuscript and in the online submission form, please provide additional information about the patient records/samples used in your retrospective study, including: a) whether all data were fully anonymized before you accessed them; b) the date range (month and year) during which patients' medical records/samples were accessed c) the source of the medical records/samples analyzed in this work (e.g. hospital, institution or medical center name).**

[Response]

Thank you for the opportunity to provide further clarification on these points. We have added the following sentences under the heading “**Ethics approval and consent.**”.

---
*All data were fully anonymized before any analysis. This post-hoc analysis was performed upon completion of the data collection in July 2018*.

--

Of note, this post-hoc analysis used a prospectively collected dataset in a multicenter cohort setting. Therefore, we have not accessed any medical records from any hospital as the dataset was already finalized and anonymized when we accessed to it.

**3. Thank you for stating the following in the Funding Section of your manuscript:**

**"This study did not receive any specific grant from funding agencies in the public, commercial, or not-for-profit sectors. RU is supported by the Masason Foundation (MF) and has received a grant from MF. The funding body played no role in the study design; data collection, management, analysis, or interpretation; manuscript preparation; or the decision to submit the report for publication."**

**We note that you have provided funding information that is not currently declared in your Funding Statement. However, funding information should not appear in the Acknowledgments section or other areas of your manuscript. We will only publish funding information present in the Funding Statement section of the online submission form.**

**Please remove any funding-related text from the manuscript and let us know how you would like to update your Funding Statement. Currently, your Funding Statement reads as follows:**

**"The authors received no specific funding for this work."**

**Please include your amended statements within your cover letter; we will change the online submission form on your behalf.**

[Response]

Thank you for your guidance for revising the Funding section for clarity. Please find the amended statements below for the online submission form:

---

*RU is supported by the Masason Foundation (MF) and has received a grant from MF. The funding body played no role in the study design; data collection, management, analysis, or interpretation; manuscript preparation; or the decision to submit the report for publication*

---

**4. In your Data Availability statement, you have not specified where the minimal data set underlying the results described in your manuscript can be found. PLOS defines a study's minimal data set as the underlying data used to reach the conclusions drawn in the manuscript and any additional data required to replicate the reported study findings in their entirety. All PLOS journals require that the minimal data set be made fully available. For more information about our data policy, please see** [**http://journals.plos.org/plosone/s/data-availability**](http://journals.plos.org/plosone/s/data-availability)**. Upon re-submitting your revised manuscript, please upload your study’s minimal underlying data set as either Supporting Information files or to a stable, public repository and include the relevant URLs, DOIs, or accession numbers within your revised cover letter. For a list of acceptable repositories, please see http://journals.plos.org/plosone/s/data-availability#loc-recommended-repositories. Any potentially identifying patient information must be fully anonymized.**

**Important: If there are ethical or legal restrictions to sharing your data publicly, please explain these restrictions in detail. Please see our guidelines for more information on what we consider unacceptable restrictions to publicly sharing data: http://journals.plos.org/plosone/s/data-availability#loc-unacceptable-data-access-restrictions. Note that it is not acceptable for the authors to be the sole named individuals responsible for ensuring data access.**

**We will update your Data Availability statement to reflect the information you provide in your cover letter.**

[Response]

Thank you for the insightful comments regarding the data sharing policy. Please find the revised statement below:

---

*Data cannot be shared publicly because of confidentiality. Data are available from the Japanese Association for Acute Medicine (JAAM) SPICE study group/Ethics Committee for researchers who meet the criteria for access to confidential data. Please contact the corresponding author and/or the ethics committee (contact via jaam-6@bz04.plala.or.jp).*

---

**Point to point response to the reviewers:**

**Reviewer #1: The study analyzes a group of patients admitted to 34 Japanese hospitals for 2 years on suspicion of infection. The value of the qSOFA scale is calculated at the time of suspected infection and the qSOFA calculation is added along with other models: 1) qSOFA; 2) qSOFA+Age; 3) q SOFA+CFS; 4) qSOFA+CCI; 5) qSOFA+lactate levels; 6) qSOFA+Age+CCI+CFS+lactate levels. The study is very interesting, but I think it presents several problems. On the one hand, they do not calculate the sample size, I consider that they do not calculate the sample size. They would have to explain how they calculate the analyzed models. On the other hand, they would have to improve the statistical analyses they perform.**

[Response]

Thank you very much for the insightful comment. Below, we will address both sample size calculation and details of the analyses.

**Introduction:**

**I think the introduction is correct.**

[Response]

Thank you very much for the supportive comment.

**Material and methods.**

**The authors would have to perform a sample size calculation. They would have to specify how they calculate each of the analyzed models.**

[Response]

This study is a post-hoc analysis of the readily collected SPICE-ER database [1]. As such, all available data (i.e. 1060 patients) on the database were used to maximize the power and generalizability of the results. After excluding 109 patients with missing data (99 for lactate, 7 for qSOFA, and 3 for CFS), 951 were included in the analysis.

There are no generally accepted approaches to estimating the sample size requirements for derivation and validation studies of risk prediction models. Some have suggested having at least 10 events per candidate variable for the derivation of a model and at least 100 events for validation studies [2]. From this point of view, our dataset meets both criteria as we have assessed at the maximum of five variables (i.e**.** qSOFA, Age, CCI, CFS, lactate) with 151 events.

**They would have to do a normality study of the quantitative variables analyzed.**

[Response]

As suggested, we have performed the Kolmogorov-Smirnov (K-S) test [3] to assess the normality of each variable.

We have edited Table 1 following the result of this test.

Table: The Result of the Kolmogorov-Smirnov Test

|  | **Name** | **D** | **P value** |
| --- | --- | --- | --- |
| **1** | Age | 0.117 | <0.001 |
| **2** | Clinical Frailty Scale | 0.137 | <0.001 |
| **3** | Charlson Index | 0.199 | <0.001 |
| **4** | qSOFA | 0.235 | <0.001 |
| **5** | Lactate | 0.221 | <0.001 |
| **6** | Respiratory Rate | 0.115 | <0.001 |
| **7** | Systolic Blood Pressure | 0.027 | 0.4936 |
| **8** | Disatolic Blood Pressure | 0.047 | 0.0318 |
| **9** | Mean Blood Pressure | 0.024 | 0.6446 |
| **10** | Heart Rate | 0.04 | 0.1024 |
| **11** | GCS – Eyes | 0.44 | <0.001 |
| **12** | GCS – Verbal | 0.271 | <0.001 |
| **13** | GCS – Motor | 0.427 | <0.001 |
| **14** | GCS | 0.295 | <0.001 |
| **15** | Body Temperature | 0.158 | <0.001 |
| **16** | Hospital Stay | 0.177 | <0.001 |

Abbreviation: GCS, Glasgow Coma Scale; SD, standard deviation; qSOFA, quick sepsis-related organ failure assessment

**They would also have to calculate in addition to sensitivity, specificity, positive and negative predictive values likehood positive and negative ratios with their respective confidence intervals at 95%**

[Results]

Thank you very much for the insightful comment. As suggested, we have amended **Table 2** below. We have calculated the 95% confidence interval of each value using the cut-off at the Youden’s index. In addition, we have repeated the same analysis in a subgroup of patients younger than 70 years old.

**Table 2. Comparison of areas under the curve and other key clinical statistics between prediction models**

| Model | AUC (95% CI) | p-value* |
| --- | --- | --- |
| qSOFA-Only | 0.627 [0.580; 0.673] | reference |
| qSOFA+Age | 0.650 [0.602; 0.698] | 0.058 |
| qSOFA+CCI | 0.639 [0.592; 0.686] | 0.109 |
| qSOFA+CFS | 0.663 [0.617, 0.710] | 0.017 |
| qSOFA+lactate | 0.695 [0.646; 0.743] | <0.001 |
| qSOFA+Age+CCI+CFS+lactate | 0.730 [0.687; 0.774] | <0.001 |

| Model | Sensitivity (95%CI) | Specificity (95%CI) | Positive predictive value(95%CI) | Negative predictive value(95%CI) | Positive likelihood ratio(95%CI) | Negative likelihood ratio(95%CI) |
| --- | --- | --- | --- | --- | --- | --- |
| qSOFA only | 0.55  ［0.47; 0.63］ | 0.63  ［0.59; 0.66］ | 0.22  ［0.18; 0.26］ | 0.88  ［0.85; 0.91］ | 1.48  ［1.24; 1.75］ | 0.72  ［0.60; 0.86］ |
| qSOFA+Age | 0.62  ［0.54; 0.70］ | 0.60  ［0.57; 0.64］ | 0.23  ［0.19; 0.27］ | 0.89  ［0.87; 0.92］ | 1.57  ［1.35; 1.82］ | 0.63  ［0.51; 0.77］ |
| qSOFA+CCI | 0.67  ［0.59; 0.74］ | 0.55  ［0.51; 0.58］ | 0.22  ［0.18; 0.26］ | 0.90  ［0.87; 0.92］ | 1.47  ［1.28; 1.68］ | 0.61  ［0.48; 0.77］ |
| qSOFA+CFS | 0.60  ［0.51; 0.68］ | 0.66  ［0.62; 0.69］ | 0.25  ［0.20; 0.29］ | 0.90  ［0.87; 0.92］ | 1.73  ［1.47; 2.03］ | 0.62  ［0.50; 0.75］ |
| qSOFA+lactate | 0.59  ［0.51; 0.67］ | 0.75  ［0.72; 0.78］ | 0.31  ［0.25; 0.36］ | 0.91  ［0.88; 0.93］ | 2.35  ［1.96; 2.81］ | 0.55  ［0.45; 0.67］ |
| qSOFA+Age+  CCI+CFS+lactate | 0.64  ［0.56; 0.72］ | 0.70  ［0.67; 0.73］ | 0.29  ［0.24; 0.34］ | 0.91  ［0.89; 0.93］ | 2.14  ［1.83; 2.51］ | 0.51  ［0.41; 0.64］ |

*p-value: comparison with the AUC of qSOFA-Only (model 1)

Key clinical statistics calculated with the Youden’s index, whereby the sum of sensitivity and specificity is the biggest. Abbreviations: AUC, area under the curve; CCI, Charlson comorbidity index; CFS, Clinical Frailty Scale; CI, confidence interval; NPV, negative predictive value; PPV, positive predictive value; qSOFA; quick sepsis-related organ failure assessment

**Table 3. Comparison of areas under the curve and other key clinical statistics between prediction models for patients aged <70 years**

| Models | AUC [95% CI] | p-value* |
| --- | --- | --- |
| qSOFA-Only | 0.579 [0.483; 0.675] | reference |
| qSOFA+Age | 0.595 [0.495; 0.695] | 0.597 |
| qSOFA+CCI | 0.591 [0.494; 0.689] | 0.600 |
| qSOFA+CFS | 0.624 [0.523; 0.724] | 0.319 |
| qSOFA+lactate | 0.718 [0.620; 0.816] | 0.025 |
| qSOFA+Age+CCI+CFS+lactate | 0.724 [0.634; 0.813] | 0.015 |

| **Model** | **Sensitivity**  **(95% CI)** | **Specificity**  **(95% CI)** | **Positive predictive value(95% CI)** | **Negative predictive value(95% CI)** | **Positive likelihood ratio(95% CI)** | **Negative likelihood ratio(95% CI)** |
| --- | --- | --- | --- | --- | --- | --- |
| qSOFA only | 0.59  ［0.49; 0.67］ | 0.61  ［0.56; 0.65］ | 0.25  ［0.20; 0.30］ | 0.87  ［0.83; 0.90］ | 1.49  ［1.24; 1.78］ | 0.68  ［0.55; 0.85］ |
| qSOFA+Age | 0.50  ［0.40; 0.59］ | 0.73  ［0.69; 0.77］ | 0.29  ［0.23; 0.36］ | 0.87  ［0.83; 0.90］ | 1.83  ［1.46; 2.29］ | 0.69  ［0.58; 0.83］ |
| qSOFA+CCI | 0.65  ［0.56; 0.73］ | 0.58  ［0.54; 0.62］ | 0.26  ［0.21; 0.31］ | 0.88  ［0.84; 0.91］ | 1.55  ［1.32; 1.83］ | 0.60  ［0.47; 0.77］ |
| qSOFA+CFS | 0.66  ［0.57; 0.74］ | 0.60  ［0.57; 0.65］ | 0.27  ［0.22; 0.32］ | 0.89  ［0.85; 0.92］ | 1.67  ［1.41; 1.96］ | 0.56  ［0.44; 0.73］ |
| qSOFA+lactate | 0.60  ［0.51; 0.69］ | 0.75  ［0.71; 0.79］ | 0.35  ［0.29; 0.42］ | 0.89  ［0.86; 0.92］ | 2.43  ［1.98; 2.98］ | 0.53  ［0.42; 0.66］ |
| qSOFA+Age+CCI  +CFS+lactate | 0.66  ［0.57; 0.74］ | 0.68  ［0.64; 0.72］ | 0.32  ［0.26; 0.38］ | 0.90  ［0.87; 0.93］ | 2.07  ［1.74; 2.47］ | 0.50  ［0.39; 0.64］ |

*p-value: comparison with the AUC of qSOFA-Only (model 1)

Key clinical statistics calculated with the Youden’s index, whereby the sum of sensitivity and specificity is the biggest. Abbreviations: AUC, area under the curve; CCI, Charlson comorbidity index; CFS, Clinical Frailty Scale; CI, confidence interval; NPV, negative predictive value; PPV, positive predictive value; qSOFA; quick sepsis-related organ failure assessment

**Results.**

**The results are adequate but the results of the sensitivity, specificity, PPV and NPV that the authors give should give them with 95% confidence intervals. They would also have to calculate positive and negative likehood ratios. Both in the global analysis and in the analysis that make the youngest population below 70 years. I am drawn to the low number of patients recruited from 32 hospitals over two years during the study, the authors should have calculated a sample size.**

[Results]

As per your suggestions and comments, we have addressed both sample size calculations and amended the statistical analyses. Details are mentioned in our response to the previous comments.

**Discussion.**

**The authors should improve the discussion by only writing about the usefulness of the qSOFA scale but do not affect the results obtained by the rest of the models studied. They would have to better explain the results obtained from each model and because the models improve, even if it is slightly to the qSOFA score.**

[Response]

We completely agree with the reviewer. We believe that a slight improvement in qSOFA may reflect the fact that qSOFA only captures physiological derangements.

In the ED, we always process and synergize various information (e.g. patient background, past histories, social status, examination findings, laboratory results, and patient wishes) to make a clinical prediction. Addition of any such variable might somehow improve a simple physiological score like qSOFA. As qSOFA focuses on acute physiological values, we chose risk scores to represent different categories of sepsis mortality: age and Charlson comorbidity score for patient demographic variables; CFS for the functional variable, and lactate for the laboratory variable.

We have added this in the **Discussion** section.

**The authors tell us that there are several studies that analyze the result of qSOFA but only analyze its results against 2 of them and especially one of them, I think the authors would have to expand the number of articles with which to compare their results.**

[Response]

We thank the reviewer for this constructive comment, and we have incorporated this suggestion throughout our **Discussion** section. Following this advice, we have performed a scoping review of the literature once again. We found additional similar literature assessing additional improvements of the qSOFA score in the ED settings. We have analyzed those results, compared them with our results, and enriched the **Discussion** section with this new information.

**Reviewer #2: This study aims to analyze a the value of the qSOFA scale in patients of suspicion of infection in a multicenter approach.**

**However, I have the feeling the data is underused from the statistical point of view and some modifications should be performed before publication.**

[Response]

Thank you very much for the insightful comment. Below, we have provided a point-to-point response to each comment.

**Major concerns,**

**- Please, confirm in methods (or not) if all the patients suffer from infection. qSOFA should be applied to those with confirmed infection since it was designed to predict infection.**

[Response]

We appreciate this opportunity for further clarification. We had included all the patients with suspected infection in the emergency department, instead of those with confirmed infection for two reasons:

First, the original study to develop qSOFA included all patients with “suspected” infection instead of “confirmed” infection [4]. Following the original study, we also included all patients with suspected infection based on the assessment of experienced emergency physicians.

Second, microbiological data is usually not available in the ED. In the clinical setting, the confirmation of the infection is based on culture results, which usually take a few days to be reported. Hence, such results are not available when clinicians use the qSOFA in the ED. For this reason, our inclusion criteria reflect similar real-world settings where qSOFA is used.

For these two reasons, we have included patients with suspected infection, instead of confirmed infection. Fortunately, our dataset included microbiological data of each patient with minimal missing data.Therefore, we have performed the subgroup analysis based on their microbiological evidence and included this in our response below.

**- I recommend to analyze those if there are differences in prediction of mortality in those with confirmed infection and those without.**

[Response]
As suggested, we have repeated the analyses based on the confirmation of infection, as defined by the microbiological evidence obtained during the admission. Of the 951 patients included in the main analysis, 5 patients did not have any data regarding their microbiological evidence. Of the 946 patients with complete data of microbiology results, 866 patients had confirmed infection.

As summarized below, each model provided similar results to the main analysis. Similar to the main results, addition of various variables only marginally improved the qSOFA in the subgroup of patients with confirmed infections. The results of those without infections were difficult to interpret, as all the models includes AUC of 0.50 in its confidence interval, which means that none of the six models work as good as a coin-toss. This cohort only includes 80 patients, which might have affected this wide confidence interval.

Above results were newly added in the Supplementary material.

**Confirmed infection (n=866)**

|  | **estimate** | **95%CI, lower** | **95%CI, upper** | **p-value** | **threshold** | **sensitivity** | **specificity** | **ppv** | **npv** |
| --- | --- | --- | --- | --- | --- | --- | --- | --- | --- |
| **qSOFA only** | 0.632 | 0.583 | 0.680 | 1.000 | 0.165 | 0.571 | 0.617 | 0.223 | 0.882 |
| **qSOFA+Age** | 0.653 | 0.603 | 0.703 | 0.096 | 0.164 | 0.614 | 0.609 | 0.232 | 0.891 |
| **qSOFA+CCI** | 0.645 | 0.597 | 0.693 | 0.106 | 0.149 | 0.636 | 0.588 | 0.229 | 0.893 |
| **qSOFA+CFS** | 0.669 | 0.621 | 0.717 | 0.018 | 0.167 | 0.607 | 0.653 | 0.252 | 0.896 |
| **qSOFA+lactate** | 0.702 | 0.653 | 0.751 | 0.000 | 0.160 | 0.600 | 0.745 | 0.312 | 0.906 |
| **qSOFA+Age+CCI+CFS+lactate** | 0.738 | 0.693 | 0.782 | 0.000 | 0.154 | 0.679 | 0.669 | 0.284 | 0.915 |

**Non-infection (n=80)**

|  | **estimate** | **95%CI, lower** | **95%CI, upper** | **p-value** | **threshold** | **sensitivity** | **specificity** | **ppv** | **npv** |
| --- | --- | --- | --- | --- | --- | --- | --- | --- | --- |
| **qSOFA only** | 0.496 | 0.330 | 0.661 | 1.000 | 0.125 | 0.800 | 0.257 | 0.133 | 0.900 |
| **qSOFA+Age** | 0.591 | 0.406 | 0.776 | 0.455 | 0.138 | 0.700 | 0.529 | 0.175 | 0.925 |
| **qSOFA+CCI** | 0.541 | 0.344 | 0.738 | 0.639 | 0.125 | 0.500 | 0.614 | 0.156 | 0.896 |
| **qSOFA+CFS** | 0.593 | 0.409 | 0.777 | 0.411 | 0.120 | 0.700 | 0.514 | 0.171 | 0.923 |
| **qSOFA+lactate** | 0.522 | 0.291 | 0.753 | 0.848 | 0.117 | 0.500 | 0.614 | 0.156 | 0.896 |
| **qSOFA+Age+CCI+CFS+lactate** | 0.617 | 0.421 | 0.813 | 0.344 | 0.142 | 0.500 | 0.714 | 0.200 | 0.909 |

**- I recommed to analyze risk factors for mortality to elucidate if qSOFA represent a variable associated with mortality.**

[Response]

As suggested, we have assessed the odds ratio of qSOFA in six different logistic regression models. As summarized below, the odds ratio of qSOFA was consistently above 1 if a model did not include lactate; however, addition of lactate decreased the odds ratio of qSOFA. Given the small variance inflation factor, the multicolinearity of the qSOFA and lactate are unlikely in our dataset. This result might be related to the sample size.

|  | **OR** | **95% CI, upper** | **95% CI, lower** | **VIF** |
| --- | --- | --- | --- | --- |
| **Model 1** |  |  |  |  |
| qSOFA | 2.06 | 1.45 | 2.92 | 1 |
| **Model 2** |  |  |  |  |
| qSOFA | 1.91 | 1.34 | 2.72 | 1.01 |
| Age | 1.03 | 1.01 | 1.04 | 1.01 |
| **Model 3** |  |  |  |  |
| qSOFA | 1.69 | 1.17 | 2.44 | 1.06 |
| Clinical Frailty Scale | 1.22 | 1.11 | 1.34 | 1.06 |
| **Model 4** |  |  |  |  |
| qSOFA | 2.07 | 1.46 | 2.95 | 1 |
| Charlson Comorbidity Index | 1.05 | 0.99 | 1.12 | 1 |
| **Model 5** |  |  |  |  |
| qSOFA | 1.43 | 0.97 | 2.09 | 1.1 |
| Lactate | 1.19 | 1.13 | 1.26 | 1.1 |
| **Model 6** |  |  |  |  |
| qSOFA | 1.09 | 0.73 | 1.64 | 1.17 |
| Age | 1.03 | 1.01 | 1.04 | 1.14 |
| Clinical Frailty Scale | 1.2 | 1.08 | 1.33 | 1.19 |
| Charlson Comorbidity Index | 1.03 | 0.96 | 1.1 | 1.04 |
| Lactate | 1.23 | 1.16 | 1.3 | 1.16 |

**Minor concerns:**

**- Disccuss more in deep your results with those studies from the literature. Expand references.**

[Response]

We thank the reviewer for this constructive comment, and we have incorporated this suggestion throughout our **Discussio**n section. Following this advice, we have performed another scoping review of the literature. We found additional similar literature assessing additional improvement of the qSOFA score in the ED settings. We have analyzed those results, compared them with our results, and enriched the **Discussion** section.

**References**

[1]	Shiraishi A, Gando S, Abe T, Kushimoto S, Mayumi T, Fujishima S, et al. Quick sequential organ failure assessment versus systemic inflammatory response syndrome criteria for emergency department patients with suspected infection. Sci Rep 2021;11:5347. doi:10.1038/s41598-021-84743-3.

[2]	Collins GS, Reitsma JB, Altman DG, Moons KGM. Transparent Reporting of a multivariable prediction model for Individual Prognosis Or Diagnosis (TRIPOD): The TRIPOD Statement. Ann Intern Med 2015;162:55. doi:10.7326/M14-0697.

[3]	Massey FJ. The Kolmogorov-Smirnov Test for Goodness of Fit. J Am Stat Assoc 1951;46:68–78. doi:10.1080/01621459.1951.10500769.

[4]	Seymour CW, Liu VX, Iwashyna TJ, Brunkhorst FM, Rea TD, Scherag A, et al. Assessment of clinical criteria for sepsis for the third international consensus definitions for sepsis and septic shock (sepsis-3). JAMA 2016;315:762–74. doi:10.1001/jama.2016.0288.
